# Homologous Recombination Is Associated with Enhanced Anti-Tumor Innate Immunity and Favorable Prognosis in Head and Neck Cancer

**DOI:** 10.3390/cancers17243999

**Published:** 2025-12-15

**Authors:** Negin Soghli, Aminollah Khormali, Aimin Peng

**Affiliations:** 1 Department of Biomedical Sciences, Adams School of Dentistry, The University of North Carolina at Chapel Hill, Chapel Hill, NC 27514, USA; nsoghli@unc.edu; 2 Adams School of Dentistry, The University of North Carolina at Chapel Hill, Chapel Hill, NC 27514, USA; khormali@unc.edu; 3 Lineberger Comprehensive Cancer Center, The University of North Carolina at Chapel Hill, Chapel Hill, NC 27514, USA

**Keywords:** head and neck squamous cell carcinoma, HNSCC, homologous recombination, HR, immune response

## Abstract

Background/Objectives: Head and neck squamous cell carcinoma (HNSCC) is an aggressive malignancy, often diagnosed at advanced stages with poor survival outcomes. Homologous recombination (HR), a major DNA double-strand break (DSB) repair pathway, safeguards genomic stability via error-free repair. While HR deficiency has been well established as a driver of genomic instability and tumorigenesis in several cancer types, the role of HR in HNSCC remains relatively understudied. Methods: Here, we analyzed the expression patterns of key HR proteins in HNSCC and investigated their association with clinical parameters, DNA methylation, immune cell infiltration, and patient survival outcome. Results: Surprisingly, our results demonstrate that HR factors are consistently upregulated in HNSCC, in both HPV-positive and HPV-negative groups. Survival analysis identified many HR factors, including ATM, BRCA1, BRCA2, PALB2, LIG1, RPA1, and RPA2, as potential prognostic biomarkers for better overall survival. Interestingly, we observed a significant correlation between HR protein overexpression and immune cell infiltration in HNSCC, suggesting a potential immunomodulatory role of HR proteins. To experimentally validate this association in both HPV-positive and -negative cell lines, we showed that MRE11 and RAD51 overexpression in HNSCC cells led to increased phosphorylation of IRF3 and STAT1, indicating activation of the cGAS/STING-mediated innate immune signaling. Conclusion: Together, our findings provide a comprehensive overview of the HR pathway in HNSCC, highlighting the dual role of HR proteins in both genomic maintenance and immune regulation. The consistent upregulation of HR proteins, their association with disease progression, and potential immunogenic effects underscore their promise as diagnostic/prognostic biomarkers and therapeutic targets in HNSCC.

## Simple Summary

Head and neck squamous cell carcinoma (HNSCC) is an aggressive malignancy characterized by limited options for early detection and persistently poor survival outcomes. The homologous recombination (HR) DNA repair pathway is a central mechanism for preserving genomic stability, but the role of HR in HNSCC remains insufficiently defined. In this study, we showed that HR factors are consistently overexpressed in HNSCC tumors, in general associations with improved patient survival, and in increased infiltration of immune effector cells. Experimental overexpression of key HR components in various HNSCC cell lines activated innate immune signaling through the cGAS–STING pathway. Together, these findings indicate that HR proteins influence both genome maintenance and tumor–immune interactions, supporting their potential utility as diagnostic and prognostic biomarkers, and informing future therapeutic strategies in HNSCC.

## 1. Introduction

Head and neck cancer (HNC) is one of the most prevalent types of cancer, with an estimated 2,041,910 new cases and 618,120 deaths projected in the world in 2025 [1]. The most common form of HNC is squamous cell carcinoma (HNSCC), accounting for over 90% of all HNC cases [2,3]. This cancer encompasses tumors arising from various sites, including the oral cavity, pharynx, tongue, mouth, paranasal sinuses, oropharynx, nasopharynx, hypopharynx, and larynx. Major risk factors of HNSCC include tobacco and alcohol, both of which contribute to genomic instability and activate oncogenic signaling pathways [2,3]. In addition, high-risk human papillomaviruses (HPVs) are responsible for a growing subset of HNSCCs in developed countries [4,5]. HPV-encoded oncoproteins disrupt key tumor suppressors to promote viral replication while compromising genomic integrity [6,7]. Clinically, HPV-positive HNSCCs are generally less aggressive and exhibit better responses to therapy than their HPV-negative counterparts [8]. The standard therapies for HNC include surgery, chemotherapy, radiotherapy, or a combination of these approaches [9]. The overall survival rate for patients with HNSCC is approximately 70–90% when diagnosed early. However, late diagnosis, which is common in HNSCC, results in a significant drop in survival rates to 40% or lower [10]. Current therapeutic methods for treating HNSCC can also cause organ dysfunction and structural damage to the swallowing and vocal cords, thereby affecting the quality of life among patients [11]. These challenges underscore the critical need for more accurate diagnostic and prognostic biomarkers to facilitate early detection and improve treatment outcomes in HNSCC.

One promising research area for discovering new cancer drug targets and biomarkers lies in the DNA damage repair pathways. Especially, double-strand breaks (DSBs) are potent inducers of genomic instability and cell death, making them crucial for both cancer progression and treatment responses [12]. The repair of DSBs is managed largely by two pathways: homologous recombination (HR) and non-homologous end joining (NHEJ) [13]. HR is a high-fidelity DNA repair pathway that plays a critical role in maintaining genome stability by accurately repairing DSBs using homologous DNA sequences. This pathway is particularly active during the S and G2 phases of the cell cycle when sister chromatids are available for repair [14]. The process begins with DSB recognition by the MRN complex (MRE11-RAD50-NBS1) which recruits and activates ATM kinase. Resection of DNA ends by CtIP generates single-stranded DNA coated by the RPA (RPA1, RPA2, RPA3) heterotrimeric complex. RPA is then replaced by RAD51 with the help of BRCA1, BRCA2, PALB2, and RAD52, enabling strand invasion into the homologous template. DNA synthesis restores the missing sequence, and final ligation by LIG 1 completes repair, preserving genomic stability. Additional factors including H2AX, MDC1, and others facilitate DNA damage signaling to promote HR [15,16].

HR deficiencies have been shown in breast, ovarian, prostate, pancreatic, and other types of cancer as an important etiological factor, consistent with the role of HR as an error-free DNA repair mechanism [17,18,19]. Genomic mutations of key HR genes cause cancer predisposition, and tumors with HR deficiencies are intrinsically sensitive to inhibition of PARP, owing to the induction of synthetic lethality [17,20]. By comparison, the involvement of HR in HNSCC is much less understood, with limited information available on the status of HR factors in HNSCC and their correlation with HNSCC progression and treatment outcomes [21]. To address these gaps, we investigated in this study the expression patterns of key HR proteins in HNSCC and their correlation with clinical outcomes, immune cell infiltration, and immune response. We assessed their potential as prognostic biomarkers that may inform personalized therapeutic options in HNSCC. In addition to these lines of data analyses, we performed functional experiments to overexpress MRE11 and RAD51 in HNSCC cells, and observed the enhanced activation of IRF3 and STAT1, suggesting a link between HR protein overexpression and innate immune activation.

## 2. Materials and Methods

### 2.1. Expression and Clinical Data Analysis

Protein expression levels for the HR pathway were analyzed using the Biocarta Pathways dataset, which includes gene–pathway associations curated from the literature (https://maayanlab.cloud/Harmonizome/dataset/Biocarta+Pathways (accessed on 20 May 2025)). The gene expression data and relevant clinical information for HNSCC patients were obtained from The Cancer Genome Atlas (TCGA) via the “TCGA gene analysis” module and the “HNSCC” dataset (https://portal.gdc.cancer.gov/ (accessed on 20 May 2025)). A *t*-test was performed to assess differences in gene expression, with a *p*-value < 0.05 considered statistically significant. All visualizations were generated using Python (version 3.13.5), specifically with libraries such as matplotlib and seaborn for plotting. Representative immunohistochemistry (IHC) images for normal and tumor tissues were obtained from The Human Protein Atlas database (https://www.proteinatlas.org/ (accessed on 20 May 2025)). In addition, the expression of proteins involved in the HR pathway was analyzed based on clinicopathological parameters such as age, gender, tumor stage, clinical stage, nodal metastasis, and clinical metastasis. A Mann–Whitney U test was used to compare protein expression levels between different groups, with a significance level set at *p*-value < 0.05.

### 2.2. Survival Analysis

Patient survival data, including gene expression levels of proteins involved in the HR pathway, were obtained from TCGA (https://portal.gdc.cancer.gov/ (accessed on 20 May 2025)) and the UCSC Xena database (https://xena.ucsc.edu (accessed on 20 May 2025)). The Kaplan–Meier method was used to estimate the survival function, with patients categorized into high- and low-expression groups based on the median expression level of the HR pathway proteins. The log-rank test was employed to compare survival distributions between these groups, with a significance level set at *α* = 0.05. In addition, the survival curves were separately conducted for HPV-positive and -negative groups, and a significance level of *α* = 0.05 was set for this experiment.

### 2.3. Correlation Analysis

For DNA methylation correlation analysis, data were retrieved from TCGA via UCSC Xena cancer browser (https://xena.ucsc.edu (accessed on 20 May 2025)), and immune cell infiltration data were retrieved from the TIMER 2.0 database (http://timer.cistrome.org/ (accessed on 10 June 2025)). Regarding the immune cell infiltration analysis, the gene expression of proteins involved in the HR pathway was analyzed in relation to immune cell infiltration levels. To assess the correlation, a Spearman’s rank correlation test was employed, which is suitable for evaluating monotonic relationships between two variables without assuming a linear relationship. The significance level was set at *p*-value < 0.05. Pearson’s correlation analysis was employed to assess the relationship between the degree of DNA methylation and gene expression level. Scatter plots were created to visualize the correlation between protein expression levels and immune cell infiltration levels for each protein in the HR pathway. Each scatter plot includes the correlation coefficient and the corresponding *p*-value, which indicate the strength and significance of the correlation, respectively.

### 2.4. Cell Culture and Transfection

Human HNSCC cell lines UM-SCC1 (SCC1) (HPV-negative) and UM-SCC38 (SCC38) (HPV-negative) were described and validated in our previous studies [22,23]; UM-SCC47 (SCC47) (HPV-positive) was previously validated by and obtained from Dr. Natalia Isaeva (UNC-Chapel Hill) [24]. As in previous publications [25,26], these cell lines were cultured in Dulbecco’s Modified Eagle Medium (DMEM) (Sigma Aldrich, MO, USA) that was supplemented with 10% fetal bovine serum (FBS) (Hyclone, Logan, UT, USA) and 100 U/mL penicillin (Corning, Phoenix, AZ, USA). Cells were maintained at 37 °C in a 5% CO_2_ atmosphere within a humidified incubator. For DNA plasmid transfection, pICE-HA-MRE11-WT (plasmid #82033, Addgene, Watertown, MA, USA) and pEGFP-RAD51 (previously described by Li et al. [27]) were used. DNA plasmid transfection was performed using the Lipofectamine LTX Reagent with PLUS Reagent (15338100, Invitrogen, Carlsbad, CA, USA) in Opti-MEM medium (31985070, Gibco, Baltimore, MD, USA) following the manufacturer’s protocol.

### 2.5. Immunoblotting

Samples were harvested using 2X Laemmli sample buffer (Bio-Rad) and resolved by sodium dodecyl sulfate-polyacrylamide gel electrophoresis (SDS-PAGE), and then transferred to nitrocellulose membranes (Millipore, Billerica, MA, USA). The membranes were blocked with 5% nonfat dry milk in 1X TBST (10 mM Tris-HCl, pH 7.5, 150 mM NaCl, 0.05% Tween 20) for 1 h and then incubated with primary antibodies overnight at 4 °C. Primary antibodies that were used included Histone H3 (1:2000, sc-517576), Phospho-Histone H2A.X (Ser139) (1:1000, sc-517348), GFP (1:1000, A01388-40), PD-L1 (1:1000, PA5-20343), Phospho-STAT1 (Tyrosine701) (1:1000, 9167), Phospho-IRF3 (S386) (1:1000, ab76493), RAD51 (1:1000, ab133534), and MRE11 (1:1000, sc-135992). After incubation with the primary antibody, the membranes were washed three times in 1X TBST, incubated with appropriate mouse and rabbit secondary antibodies (Invitrogen, Carlsbad, CA, USA) for 1 h and washed three times with 1X TBST and then detected using an enhanced chemiluminescence (ECL) substrate and a ChemiDoc Go Imaging System (Bio-Rad Laboratories, Hercules, CA, USA).

## 3. Results

### 3.1. Upregulation of HR Factors in HNC

**Figure 1:**
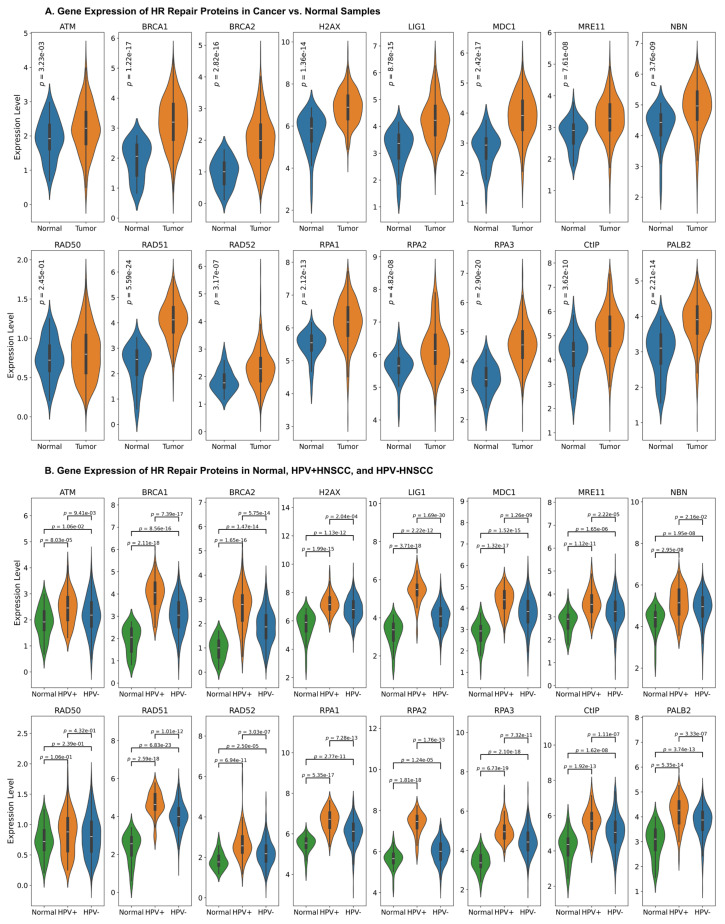
**Elevated expression of HR proteins in HNSCC.** (**A**) Comparison of the expression of HR proteins in normal vs. HNSCC patient samples. The blue color indicates normal, and the red color indicates tumor. A *t*-test was conducted to investigate the difference between gene expressions of HR repair proteins in cancer and normal tissue. The expression of all HR proteins (except for RAD50) is significantly higher in tumor samples compared to that in the normal samples. (**B**) Comparison of expression of HR genes in normal, HPV + HNSCC, and HPV-HNSCC. The green color indicates the normal group, the red color indicates the HPV+, and then blue color indicates the HPV− cases. A *t*-test was conducted to investigate the difference between the gene expression levels of each of the two groups, and a significance level of *p*-value > 0.05 was set. The expressions of all HR protein genes are significantly higher in the HPV+ group compared to those in the normal and the HPV-samples.

**Figure 2:**
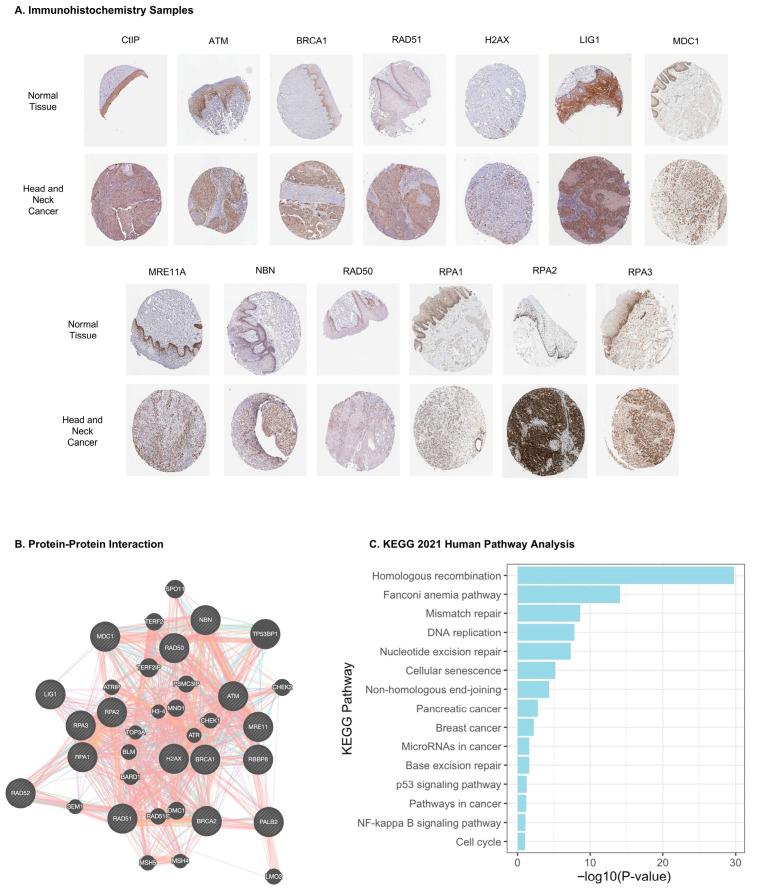
**HR protein expression and protein–protein interaction in HNSCC.** (**A**) Representative immunohistochemistry images of the genes involved in the HR repair pathway in normal and HNSCC tissue. The protein levels of CtIP, BRCA1, RAD51, H2AX, LIG1, MRE11, NBN, RPA1, RPA2, and RPA3 proteins involved in the HR repair pathway were higher in HNC tissue samples compared to those in the normal tissue (Human Protein Atlas). (**B**) Protein–protein interaction (PPI) network of HR proteins and their 20 most frequently altered neighboring genes (GeneMANIA dataset was utilized to construct the PPI network). (**C**) Enrichment analysis of HR proteins, represented as a bar plot of significantly enriched KEGG pathways.

The transcript levels of sixteen proteins involved in the HR repair pathway were examined in normal and HNSCC tissue samples using gene expression data obtained from the TCGA database, as shown in Figure 1A. The transcript levels of nearly all proteins involved in the HR repair pathway were significantly upregulated in HNSCC samples compared to normal tissue (*p*-value < 0.05). Furthermore, the difference in the expression levels of HR protein genes were examined between the normal, HPV-positive, and HPV-negative samples. The data suggests that the expression of HR protein genes was significantly higher in both HPV-negative and HPV-positive cases, compared to normal samples, with HPV-positive cases exhibiting the highest expression (Figure 1B). Additionally, the Human Protein Atlas database was used to evaluate protein expression in head and neck cancer tissue samples and normal tissues via immunohistochemistry assays, as in Figure 2A. The available immunohistochemistry staining illustrated higher expression levels of HR proteins in HNSCC tissue samples compared to normal tissues, consistent with the transcriptomic data. In addition, the evaluation of links between HR repair protein gene expression and the clinicopathological parameters indicated that LIG1, RPA2, ATM, BRCA2, NBN, and RAD50 showed significant differences across the T stages, while H2AX, MRE11, RPA1, MDC1, NBN, BRIP1, and RPA2 were differentially expressed across the N stages of HNSCC (Figures S1 and S2). No significant changes were observed between M0 and M1 stages as shown in Figure S3. Several HR proteins, including RAD51, RPA3, LIG1, RPA2, BRCA1/2, BRIP1, MDC1, RAD52, H2AX, and RPA1, were significantly upregulated in stage IV compared to those in earlier stages, whereas APEX1 was downregulated (Figure S4). Together, these findings indicate that HR pathway components are broadly upregulated in HNSCC, exhibiting HPV-, stage-, and node-associated expression patterns.

### 3.2. HR Protein Interaction Network and Pathway Enrichment Analyses

Understanding the intricate protein–protein interactions is crucial for elucidating the complex biological processes that govern cellular functions and disease mechanisms. We utilized the GeneMANIA database (https://genemania.org/ (Accessed on 20 May 2025)) database to construct the interaction network between HR proteins and their associated genes. We focused on the following proteins: TP53BP1, TERF2IP, SPO11, DMC1, MND1, TERF2, PSMC3IP, CHEK2, RAD51C, LMO2, BARD1, BLM, ATRIP, CHEK1, MSH5, ATR, SEM1, MSH4, TOP3A, and H3-4. The resulting protein interactions are depicted in Figure 2B. Furthermore, to explore the biological functions of HR proteins, we conducted a Kyoto Encyclopedia of Genes and Genomes (KEGG) pathway analysis. Beyond the HR repair pathway, these proteins were found to be implicated in several other pathways, with significant *p*-values observed for the Fanconi anemia pathway (*p*-value = 7.84 × 10^−15^), mismatch repair (*p*-value = 2.4 × 10^−9^), DNA replication (*p*-value = 1.58 × 10^−8^), nucleotide excision repair (*p*-value = 4.77 × 10^−8^), cellular senescence (*p*-value = 2.42 × 10^−4^), non-homologous end joining (*p*-value = 4.66 × 10^−5^), pancreatic cancer (*p*-value = 1.65 × 10^−3^), breast cancer (*p*-value = 6.02 × 10^−3^), micro-RNAs in cancer (*p*-value = 2.49 × 10^−2^), and base excision repair (*p*-value = 2.60 × 10^−2^) (Figure 2C). Although KEGG enrichment was dominated by DNA repair pathways, the modest enrichment of NF-κB signaling pathways aligns with the emerging concept that HR deficiency indirectly activates innate immunity. Collectively, our findings underscore the multifaceted roles of HR proteins in various biological processes and pathways.

### 3.3. The Prognostic Value of HR Proteins Expression for HNC Patients

**Figure 3:**
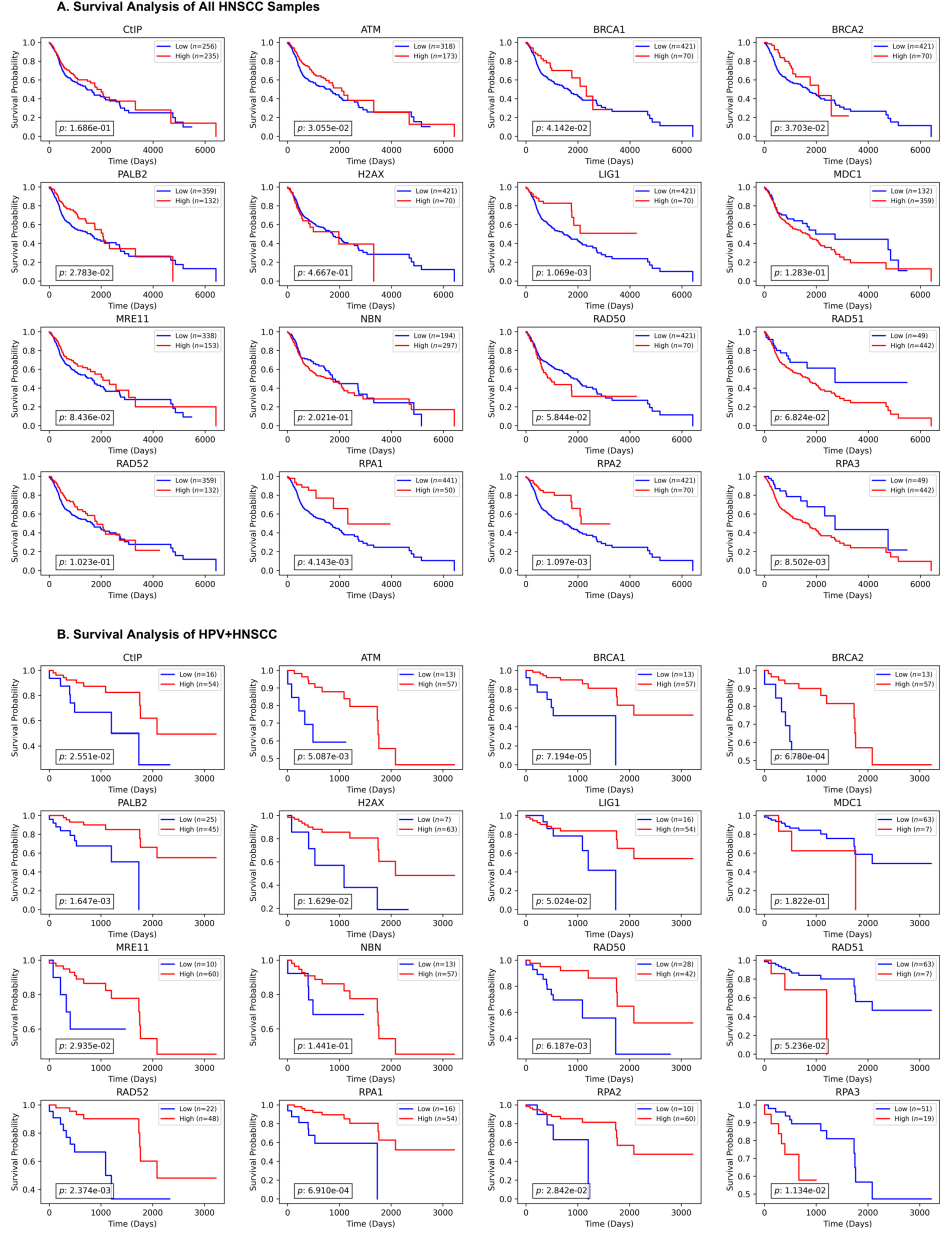
**Association of HR protein expression and patient survival.** (**A**) The prognostic value of HR proteins in HNSCC patients. Patient samples were stratified into two groups based on different quantile levels of the proposed biomarker and gene expression data. Overall survival (OS) information was obtained from TCGA database. Kaplan–Meier analysis was used to assess the survival rate, and a log-rank test was used to compare the high- and low-expression groups. The *p*-value was considered significant at *p*-value < 0.05. (**B**) The survival analysis was conducted separately for the HPV+ samples. A log-rank test was utilized to compare the high and low expressions for each set of genes with a *p*-value set at 0.05 as the significance level.

Overall survival (OS) serves as a key indicator of both therapeutic benefit and the clinical relevance of target genes in cancer progression [28]. In this study, we used the Cox proportional hazard model for analyzing the impact of HR gene overexpression on the clinical outcome of 530 HNSCC patients. The results for the Kaplan–Meier overall survival curves are shown in Figure 3A. The results indicated that the overexpression of ATM (*p*-value = 0.026), BRCA1 (*p*-value = 0.011), BRCA2 (*p*-value = 0.027), PALB2 (*p*-value = 0.014), LIG1 (*p*-value = 0.001), RPA1 (*p*-value = 0.008), and RPA2 (*p*-value < 0.001) were significantly associated with longer overall survival in HNSCC patients (Figure 2A). Thus, overexpression of specific HR proteins, including ATM, BRCA1, BRCA2, PALB2, LIG1, RPA1, and RPA2 is associated with better survival outcomes in HNSCC patients. Moreover, survival analysis was conducted for the HPV-positive samples separately, and the results indicate that a higher expression of almost all HR protein genes, except for MDC1, NBN, RAD51, and RPA3, significantly correlated with better survival rates in HNSCC patients. These data are presented in Figure 3B.

### 3.4. Correlation Between HR Proteins Expression and DNA Methylation

**Figure 4:**
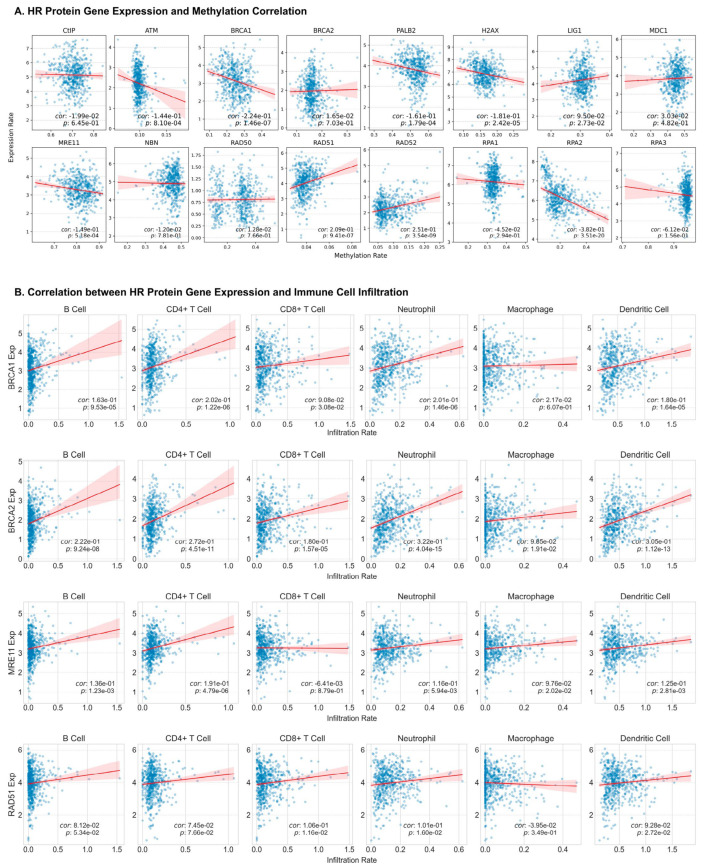
**Associations of HR protein expression and DNA methylation and immune cell infiltration**. (**A**) Correlation between DNA methylation and gene expression levels. The plots display the relationship between average DNA methylation (x-axis) and gene expression (y-axis) in HNSCC cases with available data from the TCGA database. Correlation was performed via Pearson’s correlation analysis and a significance level of *p*-value < 0.05 was set. (**B**) Correlations between immune cell infiltration and the expression of HR proteins. Spearman’s correlation test was used, and the significance level was set at *p*-value < 0.05. Dot plots of the correlation between immune cell infiltration and some HR protein gene expressions (BRCA1, BRCA2, MRE11, and RAD51).

Epigenetic alterations, particularly DNA methylation, play a critical role in cancer initiation and progression. Hypermethylation of promoter or coding regions is frequently associated with suppression of tumor suppressor gene expression. Conversely, Eden et al. [29] indicated that a decrease in DNA methylation is correlated with increased genomic stability that can be considered as a hallmark of cancer development. Here, we sought to reveal the correlation between the gene expression and DNA methylation of HR factors. The scatter plots are provided in Figure 4A. Our analysis demonstrated distinct and statistically significant correlation patterns between gene expression and promoter methylation levels among nine HR proteins. As expected, the expressions of ATM, BRCA1, PALB2, H2AX, MRE11, and RPA2 were negatively correlated with the DNA promoter region methylation. However, higher expressions of RAD51, RAD52, and LIG1 were positively correlated with DNA methylation. Thus, these results suggest that the deregulation of expression among HR proteins can be partially attributed to DNA methylation of promoter regions, whereas additional mechanisms at transcriptional and post-transcriptional levels are likely involved. Our analysis was restricted to promoter-level β values derived from TCGA tumor datasets; the methylation–expression relationships reported here should be interpreted as preliminary. Promoter methylation alone may not fully capture the epigenetic regulation of HR genes, as additional elements such as enhancer methylation, chromatin accessibility, and histone modifications can influence transcription [30]. A more comprehensive epigenetic assessment will be needed to determine the causal contribution of methylation to HR gene dysregulation in HNSCC.

### 3.5. Correlation Between HR Proteins and Immune Cell Infiltration in HNC Patients

The Tumor Immune Estimation Resource (TIMER) enables assessment of immune cell infiltration across multiple cancer types, including HNSCC, based on TCGA profiles. Our TIMER analysis revealed significant correlations between HR protein expression and immune cell infiltration in HNSCC, highlighting a complex interplay between DNA repair processes and immune surveillance. Specifically, the expression levels of ATM, BRCA1, BRCA2, CtIP, H2AX, LIG1, MDC1, MRE11, RAD52, RPA1, and RPA2 were positively correlated with B-cell infiltration (all *p*-values < 0.05). Furthermore, ATM, BRCA2, NBN, and RPA2 showed a positive correlation with the infiltration of CD4+ T-cells, CD8+ T-cells, neutrophils, macrophages, and dendritic cells. Notably, BRCA1, PALB2, and LIG1 were also positively associated with increased CD4+ and CD8+ T-cell, neutrophil, and dendritic cell infiltration (Figure 4B). MRE11 expression was positively linked to the presence of all immune cell types except CD8+ T-cells, where the correlation did not reach statistical significance. The expression of RAD50 was found to be positively associated with CD4+ T-cells, neutrophils, macrophages, and dendritic cells, although no significant correlation was observed with B-cells and CD8+ T-cells. Interestingly, RAD51 expression was specifically positively correlated with CD8+ T-cells, neutrophils, and dendritic cells, while RAD52 expression was linked to higher B-cell and CD4+ T-cell infiltration (Figure 4B). In conclusion, our findings point out the importance of HR proteins in modulating immune cell infiltration in the tumor microenvironment.

### 3.6. Correlation Between HR Proteins Gene Expression and cGAS/STING Pathway Downstream Protein Gene Expression

**Figure 5:**
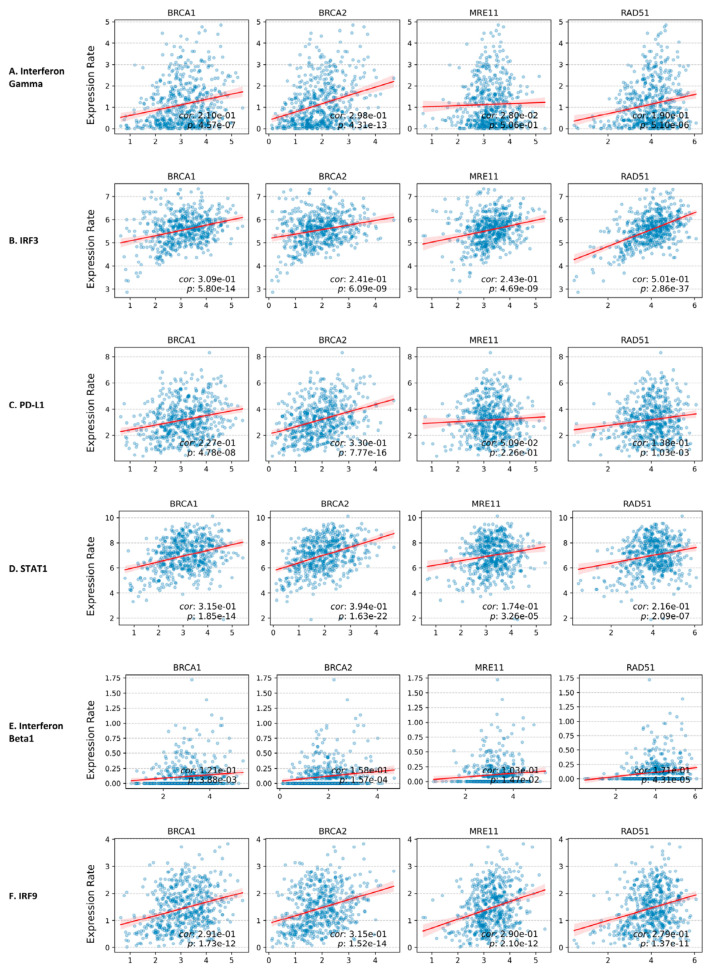
**Expression of HR proteins activate anti-tumor innate immunity.** (**A**) Co-expression of HR protein and interferon gamma. (**B**) Co-expression of HR proteins and IRF3. (**C**) Co-expression of HR proteins and PD-L1. (**D**) Co-expression of HR proteins and STAT1. (**E**) Co-expression of HR proteins and Interferon Beta1. (**F**) Co-expression of HR Proteins with IRF9.

Cytoplasmic DNA is often indicative of cellular distress, including extensive DNA damage commonly observed in cancer. Under such conditions, the cyclic GMP–AMP synthase (cGAS), typically sequestered within the nucleus, detects cytoplasmic DNA and becomes activated. Upon activation, cGAS catalyzes the synthesis of cyclic GMP–AMP (cGAMP) from nucleotides. This secondary messenger subsequently activates the stimulator of interferon genes (STING) pathway, leading to the induction of interferon production and the initiation of antitumor immune responses [31]. We investigated the co-expression patterns between HR proteins and downstream effectors of the cGAS–STING pathway to assess whether HR protein expression influences the expression of cGAS–STING signaling components. Our analysis revealed that the elevated expression of HR proteins is positively associated with increased expression of downstream proteins in the cGAS–STING pathway, including interferon gamma (Figure 5A), IRF3 (Figure 5B), PD-L1 (Figure 5C), STAT1 (Figure 5D), Interferon Beta1 (Figure 5E), and IRF9 (Figure 5F).

### 3.7. Activation of the cGAS/STING-Mediated Signaling Pathway by Upregulation of HR Factors

**Figure 6:**
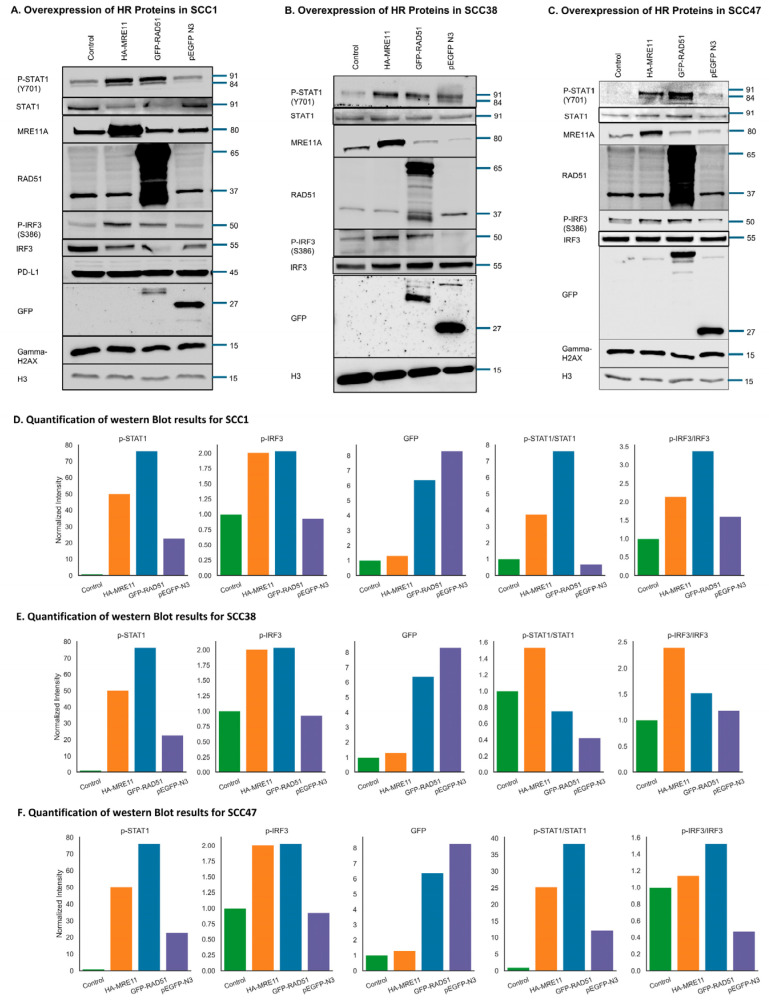
(**A**–**C**) SCC1, SCC38, and SCC47 cells were transfected with pICE-HA-MRE11, GFP-RAD51, and empty GFP vector, respectively, and then the cells were harvested 24 h post-transfection. These samples were then analyzed through immunoblotting. Activation of IRF3 and STAT1 is shown following MRE11 and RAD51 overexpression. (**D**–**F**) Quantification of Western blot results from panel A to C.

Our database analyses suggested that HR proteins may modulate anti-tumor immunity through the cGAS/STING pathway. To directly test this hypothesis, we conducted functional studies in HNSCC cell lines (SCC1, SCC38, SCC47). Interestingly, overexpression of HR proteins MRE11 and RAD51 led to robust activation of innate immune signaling, as evidenced by increased phosphorylation of IRF3 (Ser386) and STAT1 (Tyr701), two established markers of cGAS/STING pathway activation (Figure 6A–C). Quantifications for the expression of GFP and phosphorylation of STAT1 and IRF3 are provided for each cell line (Figure 6D,E). Consistent results were seen in all three cell lines, of which SCC1 and SCC38 are HPV-negative, whereas SCC47 is an HPV-positive cell line (Figure 6F). The unprocessed and uncropped scans of the Western blot images are provided in Figure S5. Thus, dysregulation of DNA repair machinery via HR protein overexpression can effectively activate downstream innate immune responses, potentially involving cytosolic DNA sensing mechanisms. This finding highlights a direct immunogenic consequence of aberrant HR activity in cancer cells.

## 4. Discussion

The accumulation of DNA damage is a pivotal driver of carcinogenesis, and the resulting genomic instability is a hallmark of cancer. To undergo malignant transformation, a cell must therefore evade mechanisms that safeguard genomic integrity. This evasion frequently occurs through the disruption of DNA damage response (DDR) pathways, including the loss of damage signaling, inactivation of cell-cycle checkpoints, or impairment of major DNA repair mechanisms [32,33]. Among various DNA lesions, double-strand breaks (DSBs) are particularly detrimental. Their inaccurate repair can lead to genomic rearrangements, such as translocations or deletions, with profound oncogenic consequences. In mammalian cells, DSBs are primarily repaired by two major pathways: NHEJ and HR. Between these two mechanisms, the HR pathway is a high-fidelity process capable of restoring the intact genomic information, using a homologous sister chromatid as a template to synthesize new DNA [34]. Consequently, HR is a crucial tumor-suppressive mechanism. This is evidenced by the cancer predisposition associated with germline mutations in BRCA1, BRCA2, and other HR genes [35,36], as well as by the frequent somatic suppression of HR observed in sporadic cancers, where it acts as a driver of tumorigenesis [37,38,39].

The HR pathway is a highly coordinated process involving multiple proteins that act sequentially to ensure the accurate repair of DSBs. Ataxia telangiectasia mutated (ATM), a phosphatidylinositol-3-kinase-like kinase (PIKK), phosphorylates histone H2AX at serine 139 to generate gamma-H2AX foci [40]. This phenomenon marks the site of damage and recruit proteins such as MDC1 and BRCA1 to repair the damage, thereby amplifying the damage signal and stabilizing the MRE11-RAD50-NBN (MRN) complex at the lesion site [41]. The MRN complex phosphorylates additional substrates such as CtIP and initiates 5′ to 3′ end resection to produce 3′ single-stranded DNA (ssDNA) overhangs [42,43]. These exposed DNA strands are rapidly coated by the replication protein A (RPA) complex to prevent degradation and recruit ATR kinase [44]. BRCA1, in cooperation with PALB2, facilitates the recruitment of BRCA2, which loads RAD51 onto RPA-coated ssDNA to form a nucleoprotein filament essential for homology search and strand invasion [45]. RAD52 serves as an additional factor that stabilizes RAD51 filaments and can mediate the annealing of complementary ssDNA [46]. Thus, it can facilitate the repair of replication-associated breaks. After the homologous template has been copied and the broken DNA ends have been resected, invaded, and extended, DNA ligase I (LIG1) seals the remaining gap to restore DNA continuity [47]. Thus, from initial detection by ATM to final ligation by LIG1, each protein operates in a tightly regulated sequence to ensure accurate and efficient homologous recombination repair, thereby preserving genomic stability.

The role of HR in HNSCC is relatively less understood. A previous study examined the expression of 84 DDR-associated genes in peripheral blood mononuclear cells from eight HNSCC patients and eight healthy controls, and reported upregulation of several HR genes, including MRE11A, RAD50, RAD51, and XRCC2 [48]. By contrast, others reported HR deficiency in HNSCC, evidenced by impaired RAD51 foci formation in HNSCC cell lines that are sensitive to PARP inhibitors [49]. In this study, we performed a comprehensive gene expression analysis to reveal the role of HR in HNSCC. Interestingly, HR genes are consistently upregulated in patients with HNSCC, despite the well-established role of HR in tumor suppression. There are several potential explanations for the pro-tumorigenesis role of HR. For example, HR upregulation may serve as a compensation and adaptive response for replication stress and increased accumulation of endogenous DSBs. Under this condition, the upregulation of HR factors helps repair these breaks at levels that allow cancer cells to survive despite genomic instability. Indeed, HNSCC cells harbor substantial levels of replication stress and endogenous DNA damage [48]. Furthermore, upregulation of HR gene expression may result from oncogenic signaling, further boosting repair capacity to sustain proliferation under stress. Along this line, RAD51 was found to be overexpressed in many solid tumors, including breast [50], lung [51], and pancreatic cancer [52]. Our findings support the therapeutic value of HR targeting in HNSCC prevention or treatment, although the development of successful interventions requires a much better understanding of how and when HR participates in HNSCC progression.

Our analyses revealed that the elevated expression of HR genes, including ATM, BRCA1, BRCA2, PALB2, LIG1, RPA1, and RPA2, was significantly associated with improved overall survival in HNSCC. These results suggest that HR pathway activity may serve as a favorable prognostic marker, which seemingly contrasts with the canonical view that HR promotes radioresistance and chemoresistance by enhancing DNA repair. Further studies are required to determine why increased HR gene expression predicts better outcomes in HNSCC. One possibility is that HR-mediated repair and adaptation to elevated replication stress and DNA damage constrain alternative error-prone repair pathways such as NHEJ, microhomology-mediated end joining (MMEJ), and single-strand annealing (SSA). In this case, tumors with HR upregulation benefit from less genomic instability and tumor aggressiveness and are more susceptible to treatments.

HPV is a small double-stranded DNA virus that depends entirely on the host cellular machinery for replication, using its proteins to manipulate cell-cycle and differentiation pathways. The viral oncoproteins E6 and E7 drive uncontrolled cell proliferation by targeting key regulators such as TP53 and RB1, and their alternatively spliced forms further enhance oncogenic potential [53]. Although E1 and E2 are required for viral replication, the loss of E2 during persistent infection removes the repression of E6 and E7, resulting in their sustained overexpression [54]. This unchecked activity promotes genomic instability and contributes to malignant transformation in cervical and head and neck cancers [55]. While our functional experiments utilized a single HPV-positive cell line with an integrated viral genome, the phosphorylation patterns of STAT1 and IRF3 following HR gene overexpression were consistent across both HPV-positive and HPV-negative models, indicating that this response is not specific to HPV status or viral genome configuration. HPV integration into the host genome is known to occur frequently in head and neck squamous cell carcinomas, although it is not required for the viral life cycle or for cellular transformation. Integrated HPV genomes have been associated with tumor recurrence and distinct transcriptional profiles, but these integrations represent only a subset of HPV-positive tumors [56]. Given that our phosphorylation results were reproducible in HPV-negative cells and not dependent on features unique to HPV-driven oncogenesis, inclusion of additional HPV-positive, episomal models would likely not have changed the mechanistic interpretation. Nevertheless, future studies incorporating both episomal and integrated HPV systems will be important to dissect whether specific integration events modify the extent of innate immune activation elicited by changes in HR gene expression.

To reveal further insights into the tumor physiology associated with HR beyond DNA repair, we investigated the anti-tumor immune response. Innate immunity plays a critical role in tumor suppression and mediates efficient treatment responses to immunotherapy, radiation, and chemotherapy. We employed the TIMER database to investigate the correlation between the expression of HR repair proteins with immune cell infiltration. The expression levels of many HR factors, especially ATM, BRCA2, and RPA2, were positively correlated with the infiltration of all immune cells including B-cells, CD4+ T-cells, CD8+ T-cells, neutrophils, macrophages, and dendritic cells. Infiltration of dendritic cells and neutrophils were particularly tightly correlated with the expression of nearly all HR repair proteins investigated in this study, except RAD52 and RPA3. Additional mechanistic studies are necessary to better understand this unexpected role of HR proteins in promoting anti-tumor immunity. Notably, a previous study showed that MRE11 facilitates the dissociation of cGAS from histone acidic patch and helps the activation of cGAS [57]. cGAS is a key sensor of cytoplasmic DNA that triggers innate immune responses against cancer and viral infections [41,42]. Activated cGAS stimulates STING at the endoplasmic reticulum, leading to type I interferon expression and inflammation [58]. Interestingly, we performed experiments to overexpress MRE11 or RAD51 in HNSCC cell lines, and observed the activation of STAT1 and IRF3, key mediators of the cGAS/ STING pathway and innate immune response. This result suggests a potentially direct causal relationship between HR and anti-tumor immunity, which may at least partially account for the observed favorable treatment response in HR-upregulated HNSCC.

## 5. Conclusions

In conclusion, our integrated bioinformatics and experimental analyses suggest that HR functions not only as a DNA damage response mechanism but also as a modulator of anti-tumor immunity, thereby influencing tumor aggressiveness and treatment outcomes in HNSCC. While these findings reveal unexpected and clinically relevant insights, several limitations should be acknowledged. First, most analyses were based on publicly available datasets, and validation in patient-derived xenografts or organoid models will be required to establish causality. Second, our observations on immune infiltration and cGAS-STING activation may not fully capture the complexity of tumor–immune interactions. Third, the mechanistic basis underlying the association between HR activity and favorable survival outcomes is not fully defined, including whether this reflects competition with error-prone DNA repair pathways and/or enhanced tumor immune responses. Building on this groundwork, future studies should prioritize mechanistic dissection and preclinical testing of HR-focused strategies, particularly in combination with radiotherapy, chemotherapy, and immune checkpoint blockades, to assess their translational potential in HNSCC.

## Data Availability

The original data presented in the study are openly available in The Cancer Genome Atlas (TCGA) at (https://portal.gdc.cancer.gov/), The Human Protein Atlas database (https://www.proteinatlas.org/), the UCSC Xena database (https://xena.ucsc.edu), and the TIMER 2.0 database (http://timer.cistrome.org/).

## Supplementary Materials

The following supporting information can be downloaded at: https://www.mdpi.com/article/10.3390/cancers17243999/s1, Figure S1: Violin plots illustrating HR protein expression levels across different clinical T stages (Stages I–IV) using TCGA data; Figure S2: Violin plots illustrating HR protein expression levels across different clinical N stages (Stages I–IV) using TCGA data; Figure S3: Violin plots illustrating HR proteins expression levels across different clinical M stages (Stages M0 and M1) using TCGA data; Figure S4: Violin plots illustrating HR proteins expression levels across different pathological stages (Stages I–IV) using TCGA data. The height of each violin reflects the range of expression values for the corresponding stage, while the central line represents the median; Figure S5: Uncropped and unprocessed scans of blotted images.

## References

[1] Siegel R.L., Kratzer T.B., Giaquinto A.N., Sung H., Jemal A. (2025). Cancer statistics, 2025. Ca.

[2] Cancer Genome Atlas Network (2015). Comprehensive genomic characterization of head and neck squamous cell carcinomas. Nature.

[3] Johnson D.E., Burtness B., Leemans C.R., Lui V.W.Y., Bauman J.E., Grandis J.R. (2020). Head and neck squamous cell carcinoma. Nat. Rev. Dis. Primers.

[4] Bosch F.X., Lorincz A., Munoz N., Meijer C.J.L.M., Shah K.V. (2002). The causal relation between human papillomavirus and cervical cancer. J. Clin. Pathol..

[5] Chaturvedi A.K., Zumsteg Z.S. (2018). A snapshot of the evolving epidemiology of oropharynx cancers. Cancer.

[6] Wallace N.A. (2020). Catching HPV in the Homologous Recombination Cookie Jar. Trends Microbiol..

[7] Holcomb A.J., Brown L., Tawfik O., Madan R., Shnayder Y., Thomas S.M., Wallace N.A. (2020). DNA repair gene expression is increased in HPV positive head and neck squamous cell carcinomas. Virology.

[8] Ang K.K., Sturgis E.M. (2012). Human Papillomavirus as a Marker of the Natural History and Response to Therapy of Head and Neck Squamous Cell Carcinoma. Semin. Radiat. Oncol..

[9] Mordzińska-Rak A., Telejko I., Adamczuk G., Trombik T., Stepulak A., Błaszczak E. (2025). Advancing Head and Neck Cancer Therapies: From Conventional Treatments to Emerging Strategies. Biomedicines.

[10] Barsouk A., Aluru J.S., Rawla P., Saginala K., Barsouk A. (2023). Epidemiology, risk factors, and prevention of head and neck squamous cell carcinoma. Med. Sci..

[11] Raber-Durlacher J.E., Brennan M.T., Verdonck-de Leeuw I.M., Gibson R.J., Eilers J.G., Waltimo T., Bots C.P., Dysphagia Section, Oral Care Study Group, Multinational Association of Supportive Care in Cancer/International Society of Oral Oncology (2012). Swallowing dysfunction in cancer patients. Support. Care Cancer.

[12] Vignard J., Mirey G., Salles B. (2013). Ionizing-radiation induced DNA double-strand breaks: A direct and indirect lighting up. Ra-diother. Oncol..

[13] Mao Z., Bozzella M., Seluanov A., Gorbunova V. (2008). DNA repair by nonhomologous end joining and homologous recombination during cell cycle in human cells. Cell Cycle.

[14] Mjelle R., Hegre S.A., Aas P.A., Slupphaug G., Drabløs F., Sætrom P., Krokan H.E. (2015). Cell cycle regulation of human DNA repair and chromatin remodeling genes. DNA Repair.

[15] Li X., Heyer W.-D. (2008). Homologous recombination in DNA repair and DNA damage tolerance. Cell Res..

[16] San Filippo J., Sung P., Klein H. (2008). Mechanism of Eukaryotic Homologous Recombination. Annu. Rev. Biochem..

[17] Konstantinopoulos P.A., Ceccaldi R., Shapiro G.I., D’ANdrea A.D. (2015). Homologous Recombination Deficiency: Exploiting the Fundamental Vulnerability of Ovarian Cancer. Cancer Discov..

[18] Nguyen L.W.M., Martens J., Van Hoeck A., Cuppen E. (2020). Pan-cancer landscape of homologous recombination deficiency. Nat. Commun..

[19] Toh M., Ngeow J. (2021). Homologous Recombination Deficiency: Cancer Predispositions and Treatment Implications. The Oncologist.

[20] Lord C.J., Ashworth A. (2017). PARP inhibitors: Synthetic lethality in the clinic. Science.

[21] Papalouka C., Adamaki M., Batsaki P., Zoumpourlis P., Tsintarakis A., Goulielmaki M., Fortis S.P., Baxevanis C.N., Zoumpourlis V. (2023). DNA Damage Response Mechanisms in Head and Neck Cancer: Significant Implications for Therapy and Survival. Int. J. Mol. Sci..

[22] Gouttia O.G., Zhao J., Li Y., Zwiener M.J., Wang L., Oakley G.G., Peng A. (2022). The MASTL-ENSA-PP2A/B55 axis modulates cisplatin resistance in oral squamous cell carcinoma. Front. Cell Dev. Biol..

[23] Wang F., Gouttia O.G., Wang L., Peng A. (2022). PARP1 Upregulation in Recurrent Oral Cancer and Treatment Resistance. Front. Cell Dev. Biol..

[24] Zhang J., Chen T., Yang X., Cheng H., Späth S.S., Clavijo P.E., Chen J., Silvin C., Issaeva N., Su X. (2018). Attenuated TRAF3 Fosters Activation of Alternative NF-κB and Reduced Expression of Antiviral Interferon, TP53, and RB to Promote HPV-Positive Head and Neck Cancers. Cancer Res..

[25] Brenner J.C., Graham M.P., Kumar B., Saunders L.M., Kupfer R., Lyons R.H., Bradford C.R., Carey T.E. (2009). Genotyping of 73 UM-SCC head and neck squamous cell carcinoma cell lines. Head Neck.

[26] Akagi K., Li J., Broutian T.R., Padilla-Nash H., Xiao W., Jiang B., Rocco J.W., Teknos T.N., Kumar B., Wangsa D. (2013). Genome-wide analysis of HPV integration in human cancers reveals recurrent, focal genomic instability. Genome Res..

[27] Li Y., Kardell M.B., Wang F., Wang L., Zhu S., Bessho T., Peng A. (2021). The Sm core components of small nuclear ribonucleo-proteins promote homologous recombination repair. DNA Repair.

[28] Driscoll J.J., Rixe O. (2009). Overall survival: Still the gold standard: Why overall survival remains the definitive end point in cancer clinical trials. Cancer J..

[29] Eden A., Gaudet F., Waghmare A., Jaenisch R. (2003). Chromosomal Instability and Tumors Promoted by DNA Hypomethylation. Science.

[30] Luo X., Li Q., Tang Y., Liu Y., Zou Q., Zheng J., Zhang Y., Xu L. (2023). Predicting active enhancers with DNA methylation and histone modification. BMC Bioinform..

[31] Dvorkin S., Cambier S., Volkman H.E., Stetson D.B. (2024). New frontiers in the cGAS-STING intracellular DNA-sensing pathway. Immunity.

[32] Kastan M.B., Bartek J. (2004). Cell-cycle checkpoints and cancer. Nature.

[33] Kennedy R.D., D’Andrea A.D. (2006). DNA Repair Pathways in Clinical Practice: Lessons From Pediatric Cancer Susceptibility Syndromes. J. Clin. Oncol..

[34] Moynahan M.E., Jasin M. (2010). Mitotic homologous recombination maintains genomic stability and suppresses tumorigenesis. Nat. Rev. Mol. Cell Biol..

[35] Chu Y.-Y., Yam C., Yamaguchi H., Hung M.-C. (2022). Biomarkers beyond BRCA: Promising combinatorial treatment strategies in overcoming resistance to PARP inhibitors. J. Biomed. Sci..

[36] Pourmasoumi P., Moradi A., Bayat M. (2024). BRCA1/2 Mutations and Breast/Ovarian Cancer Risk: A New Insights Review. Reprod. Sci..

[37] Arranz-Ledo M., Infante M., Lastra E., Olaverri A., Orozco M., Mateo L.C., Martínez N., Hernández L., Durán M. (2025). Genetic Features of Tumours Arising in the Context of Suspected Hereditary Cancer Syndromes with RAD50, RAD51C/D, and BRIP1 Germline Mutations, Results of NGS-Reanalysis of BRCA/MMR-Negative Families. Genes.

[38] Liu Y., Lin Z., Yan J., Zhang X., Tong M.-H. (2024). A Rad50-null mutation in mouse germ cells causes reduced DSB formation, abnormal DSB end resection and complete loss of germ cells. Development.

[39] Infante M., Arranz-Ledo M., Lastra E., Olaverri A., Ferreira R., Orozco M., Hernández L., Martínez N., Durán M. (2023). Profiling of the genetic features of patients with breast, ovarian, colorectal and extracolonic cancers: Association to CHEK2 and PALB2 germline mutations. Clin. Chim. Acta.

[40] Lee J.-H., Paull T.T. (2021). Cellular functions of the protein kinase ATM and their relevance to human disease. Nat. Rev. Mol. Cell Biol..

[41] Stucki M., Jackson S.P. (2006). γH2AX and MDC1: Anchoring the DNA-damage-response machinery to broken chromosomes. DNA Repair.

[42] Lamarche B.J., Orazio N.I., Weitzman M.D. (2010). The MRN complex in double-strand break repair and telomere maintenance. FEBS Lett..

[43] Takeda S., Nakamura K., Taniguchi Y., Paull T.T. (2007). Ctp1/CtIP and the MRN Complex Collaborate in the Initial Steps of Ho-mologous Recombination. Mol. Cell.

[44] Liang C.-C., Greenhough L.A., Masino L., Maslen S., Bajrami I., Tuppi M., Skehel M., Taylor I.A., West S.C. (2024). Mechanism of single-stranded DNA annealing by RAD52–RPA complex. Nature.

[45] Powell S.N., Kachnic L.A. (2003). Roles of BRCA1 and BRCA2 in homologous recombination, DNA replication fidelity and the cellular response to ionizing radiation. Oncogene.

[46] Lok B.H., Powell S.N. (2012). Molecular Pathways: Understanding the Role of Rad52 in Homologous Recombination for Therapeutic Advancement. Clin. Cancer Res..

[47] Sallmyr A., Rashid I., Bhandari S.K., Naila T., Tomkinson A.E. (2020). Human DNA ligases in replication and repair. DNA Repair.

[48] Psyrri A., Gkotzamanidou M., Papaxoinis G., Krikoni L., Economopoulou P., Kotsantis I., Anastasiou M., Souliotis V. (2021). The DNA damage response network in the treatment of head and neck squamous cell carcinoma. ESMO Open.

[49] Heitmann J., Geeleher P., Zuo Z., Weichselbaum R.R., Vokes E.E., Fetscher S., Seiwert T.Y. (2014). Poly (ADP-ribose) polymerase inhibitor efficacy in head and neck cancer. Oral Oncol..

[50] Liu Y.-C., Shen J. (2025). Meta-analysis of the association between overexpression of RAD51 family genes and prognosis and clinical features in breast cancer. Sci. Rep..

[51] Saviozzi S., Ceppi P., Novello S., Ghio P., Iacono M.L., Borasio P., Cambieri A., Volante M., Papotti M., Calogero R.A. (2009). Non–Small Cell Lung Cancer Exhibits Transcript Overexpression of Genes Associated with Homologous Recombination and DNA Replication Pathways. Cancer Res..

[52] Maacke H., Jost K., Opitz S., Miska S., Yuan Y., Hasselbach L., Lüttges J., Kalthoff H., Stürzbecher H.-W. (2000). DNA repair and recombination factor Rad51 is over-expressed in human pancreatic adenocarcinoma. Oncogene.

[53] Walline H.M., Goudsmit C.M., McHugh J.B., Tang A.L., Owen J.H., Teh B.T., McKean E., Glover T.W., Graham M.P., Prince M.E. (2017). Integration of high-risk human papillomavirus into cellular cancer-related genes in head and neck cancer cell lines. Head Neck.

[54] Pinatti L., Walline H., Carey T. (2018). Human papillomavirus genome integration and head and neck cancer. J. Dent. Res..

[55] Wiest T., Schwarz E., Enders C., Flechtenmacher C., Bosch F.X. (2002). Involvement of intact HPV16 E6/E7 gene expression in head and neck cancers with unaltered p53 status and perturbed pRb cell cycle control. Oncogene.

[56] Zur Hausen H. (2009). Papillomaviruses in the causation of human cancers—A brief historical account. Virology.

[57] Cho M.-G., Kumar R.J., Lin C.-C., Boyer J.A., Shahir J.A., Fagan-Solis K., Simpson D.A., Fan C., Foster C.E., Goddard A.M. (2024). MRE11 liberates cGAS from nucleosome sequestration during tumorigenesis. Nature.

[58] Sun L., Wu J., Du F., Chen X., Chen Z.J. (2013). Cyclic GMP-AMP Synthase Is a Cytosolic DNA Sensor That Activates the Type I Interferon Pathway. Science.

