# Supplementary material for: Homologous Recombination Is Associated with Enhanced Anti-Tumor Innate Immunity and Favorable Prognosis in Head and Neck Cancer
Source: Cancers (Basel). 2025 Dec 15;17(24):3999. doi: 10.3390/cancers17243999 (PMC12730537; doi:10.3390/cancers17243999)
Supplement: Supplementary file 1 [file cancers-17-03999-s001.zip › cancers-4030781-supplementary.pdf]

cancers MDPI

Supplementary Material

Figure S1–S4. The link between the HR gene expression and clinicopathological parameters in HNSCC.

Using the data from the TCGA database for HNSCC patients, we performed an ANOVA test to compare the gene expression levels of HR proteins between patients grouped according to clinicopathological parameters such as T stage, N stage, M stage, and clinical stage. The relevant information and violin plot are provided in Fig. S1 to S4. The results indicate that the expressions of LIG1 (p-value = 4.96×10^{-2}) and RPA2 (p-value = 2.79×10^{-2}) were significantly higher in T2 compared to those in the T3 stage. In addition, the expression of ATM was lower in T1 compared to that in T4 (p-value = 2.84×10^{-2}) as well as in T2 compared to that in the T3 (p-value = 1.49×10^{-2}) and T4 (p-value = 3.32×10^{-3}) stages. Moreover, BRCA2 was overexpressed in HNSCC patients in the T4 stage of cancer compared to that in both the T1 (p-value = 4.16×10^{-2}) and T2 (p-value = 4.70×10^{-2}) stages. Both NBN (p-value = 1.45×10^{-2}) and RAD50 (p-value = 1.32×10^{-2}) were also expressed in higher levels in the T4 stage compared to that in the T2 stage. These results are depicted in Figure S1. The expression levels of all HR proteins showed no significant difference between the M0 and M1 stages of HNSCC (Fig. S3). Regarding the difference in protein expression in different N stages of HNSCC that is presented in Figure S2, H2AX (p-value = 4.97×10^{-2}), MRE11 (p-value = 3.53×10^{-2}), and RPA1 (p-value = 2.04×10^{-2}) were significantly overexpressed in the N3 stage of HNSCC compared to those in the N1 stage. Additionally, MDC1 (p-value = 4.95×10^{-2}) and NBN (p-value = 2.80×10^{-2}) had significantly lower expression levels in the N0 stage of HNSCC compared to those in the N3 stage. The expression of BRIP1 (N0 compared to N3; p-value = 8.98×10^{-3}, and N1 compared to N3; p-value = 1.31×10^{-2}, respectively) and RPA2 (N0 compared to N3; p-value = 3.45×10^{-2}, and N1 compared to N3; p-value = 3.84×10^{-2}, respectively) was significantly increased in the N3 stage compared to that in both the N0 and N1 stages. As the clinical stage of HNSCC progresses, the gene expression levels of several HR proteins are significantly increased compared to the lower stages (Fig. S4.). For instance, the expression of RAD51 (stage I compared to stage IV; p-value = 3.90×10^{-2}, stage II compared to stage IV; p-value = 3.84×10^{-3}, and stage III compared to stage IV; p-value = 1.59×10^{-4}, respectively) and RPA3 (stage I compared to stage IV; p-value = 2.03×10^{-2}, stage II compared to stage IV; p-value = 2.47×10^{-3}, and stage III compared to stage IV; p-value = 2.85×10^{-5}, respectively) is significantly higher in patients with stage IV cancer compared to patients in all the other three stages of HNSCC, while the difference in the expression of LIG1 (stage II compared to stage IV; p-value = 5.27×10^{-3}, and stage III compared to stage IV; p-value = 2.68×10^{-5}, respectively) and RPA2 (stage II compared to stage IV; p-value = 1.39×10^{-2}, and stage III compared to stage IV; p-value = 1.25×10^{-2}, respectively) was only significantly different in stage II and III compared to that in stage IV. Up-regulation of protein expression was also observed in stage IV compared to that in stage III of HNSCC for BRCA1 (p-value = 3.38×10^{-3}), BRCA2 (p-value = 1.37×10^{-3}), BRIP1 (p-value = 2.59×10^{-2}), H2AX (p-value = 4.24×10^{-2}), MDC1 (p-value = 1.31×10^{-2}), RAD52 (p-value = 2.32×10^{-2}), and RPA1 (p-value = 4.59×10^{-2}), while the expression of APEX1 (p-value = 1.47×10^{-2}) was significantly lower in stage I compared to that in stage IV of HNSCC. In summary, the overexpression of HR proteins in later stages of HNSCC suggests enhanced DNA repair capabilities, potential resistance to standard treatments, and the need for targeted therapies to improve patient outcomes.

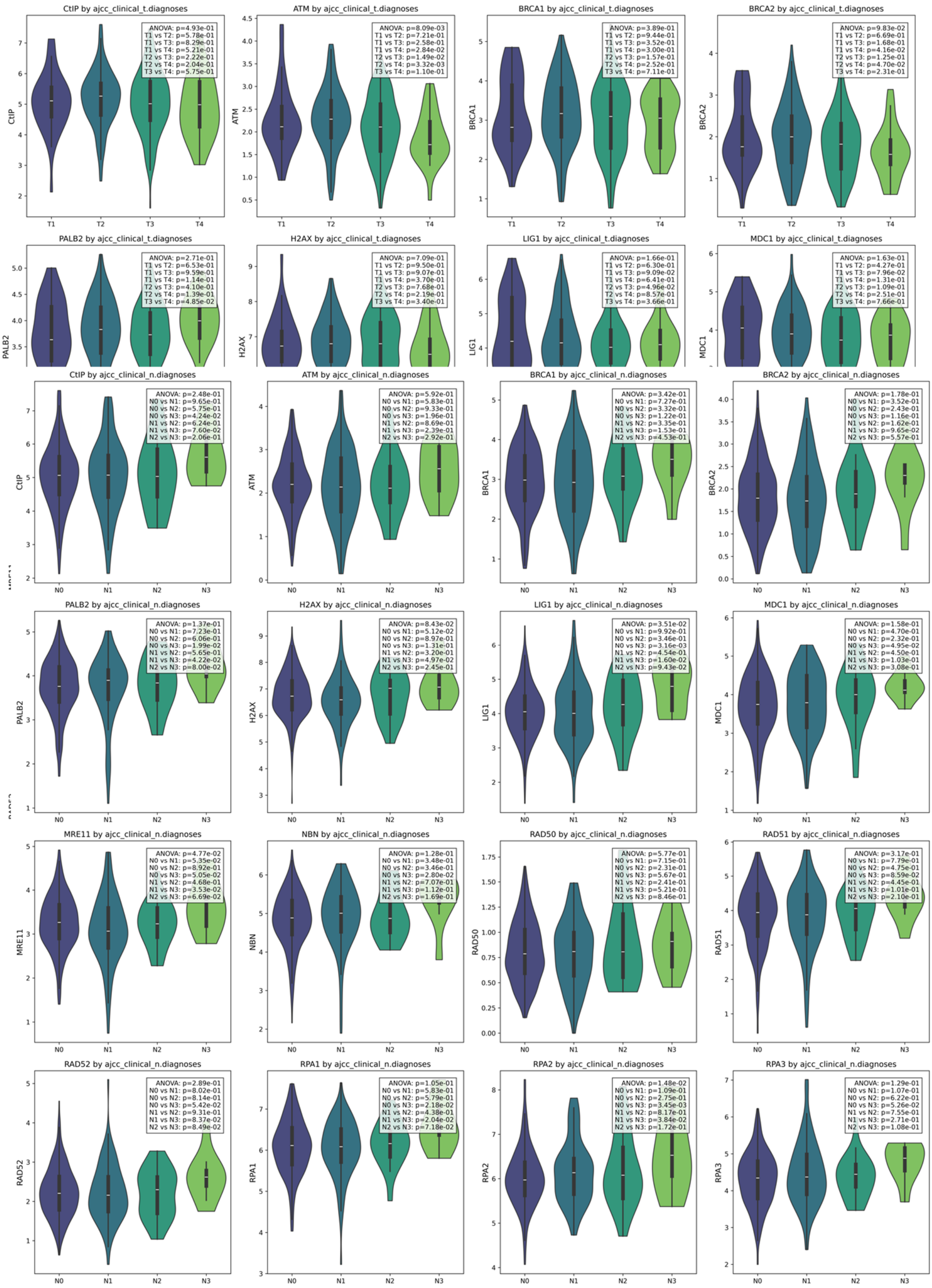

Figure S1. Violin plots illustrating HR protein expression levels across different clinical T stages (stages I -IV) using TCGA data.

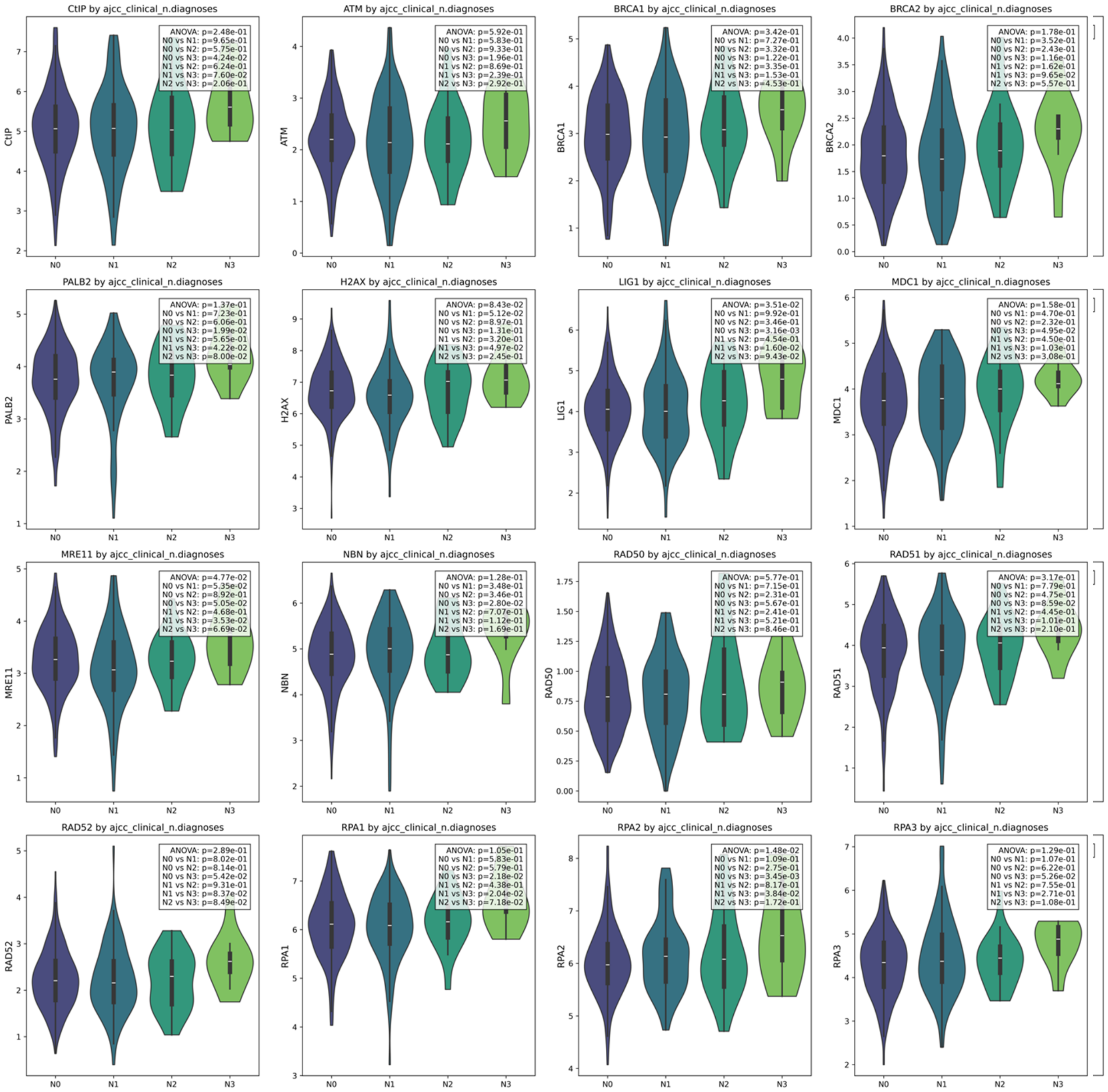

Figure S2. Violin plots illustrating HR protein expression levels across different clinical N stages (stages I -IV) using TCGA data.

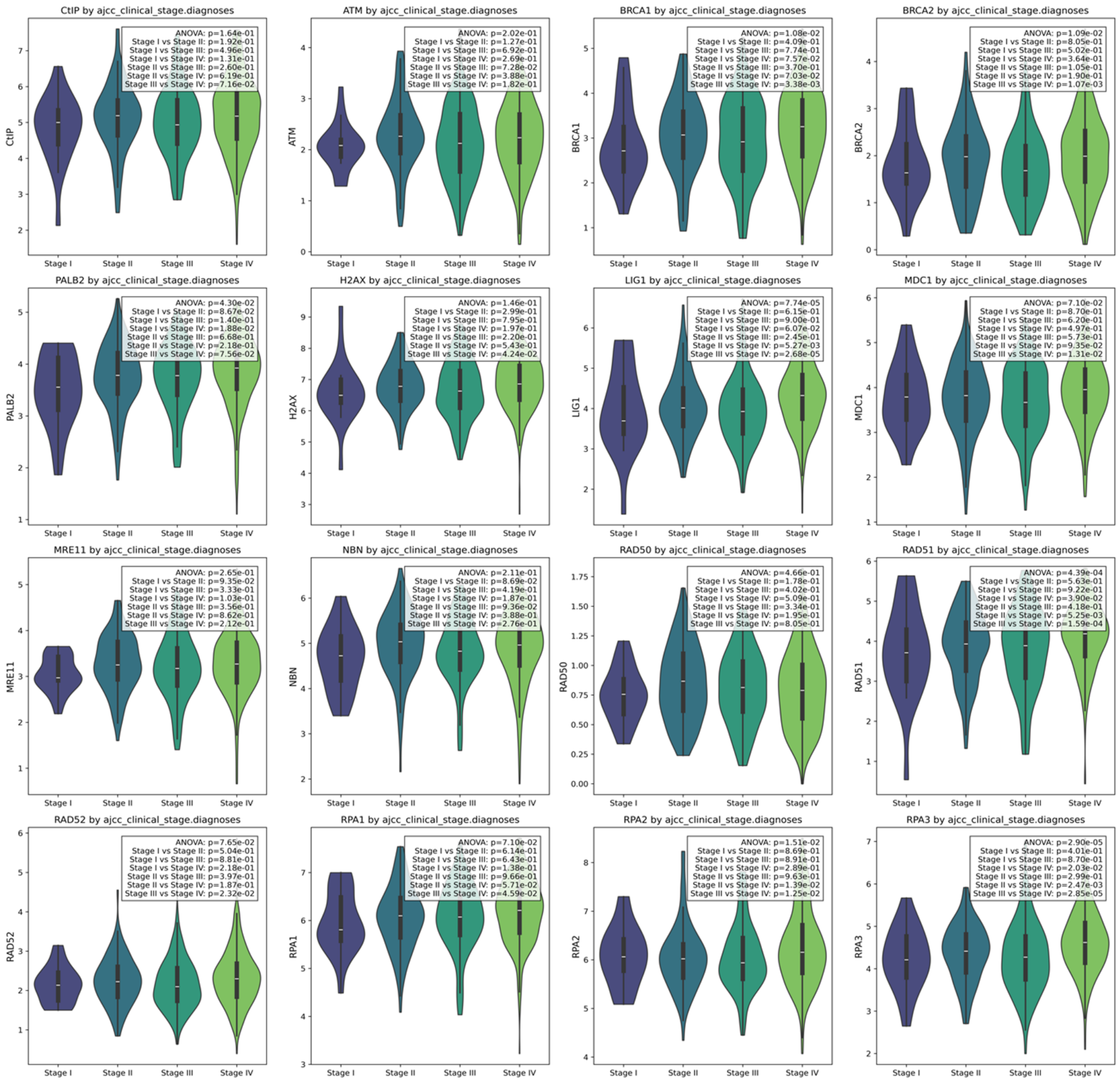

Figure S3. Violin plots illustrating HR protein expression levels across different clinical M stages (stages M0 and M1) using TCGA data.

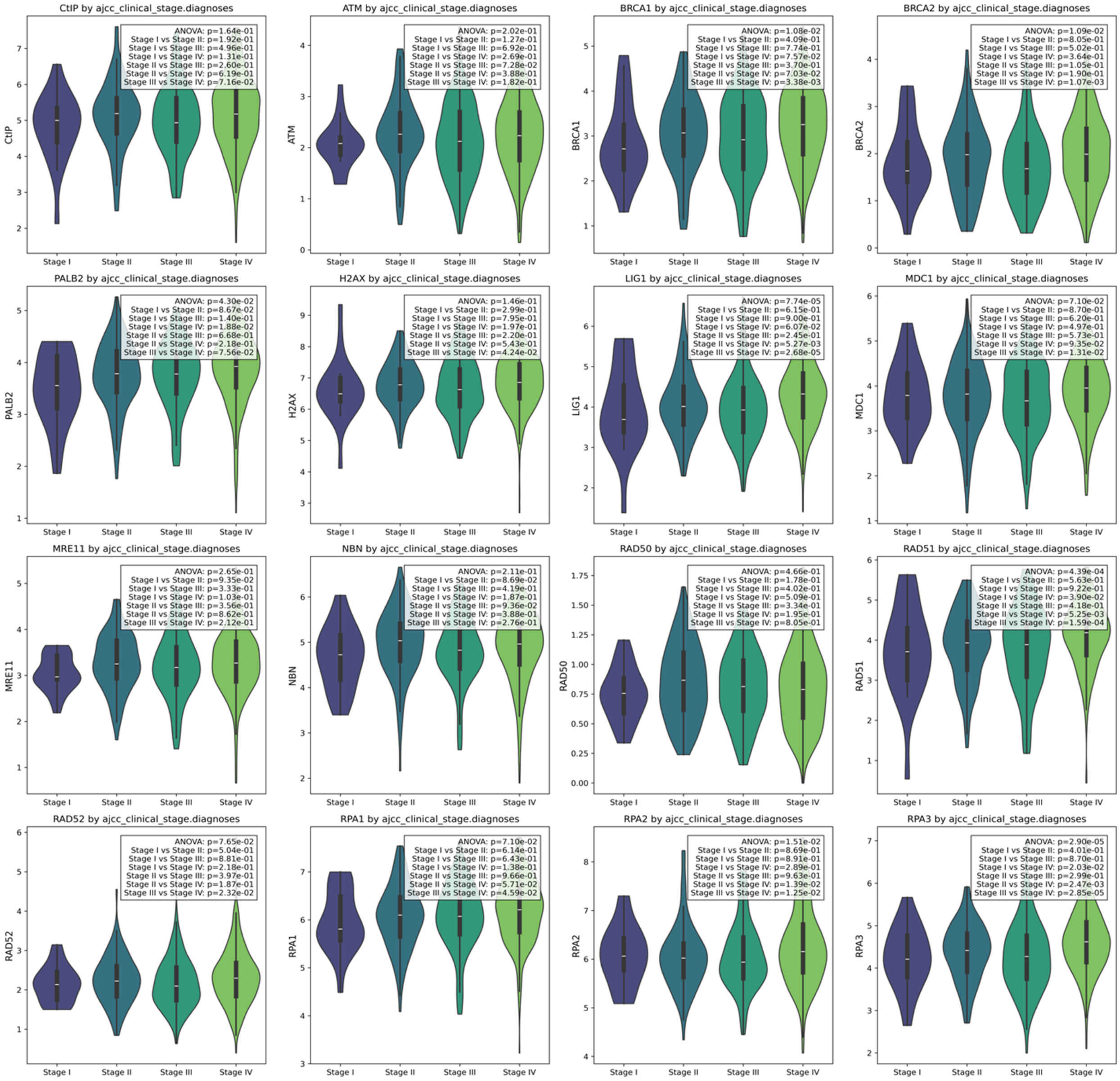

Figure S4. Violin plots illustrating HR protein expression levels across different pathological stages (stages I - IV) using TCGA data. The height of each violin reflects the range of expression values for the corresponding stage, while the central line represents the median.

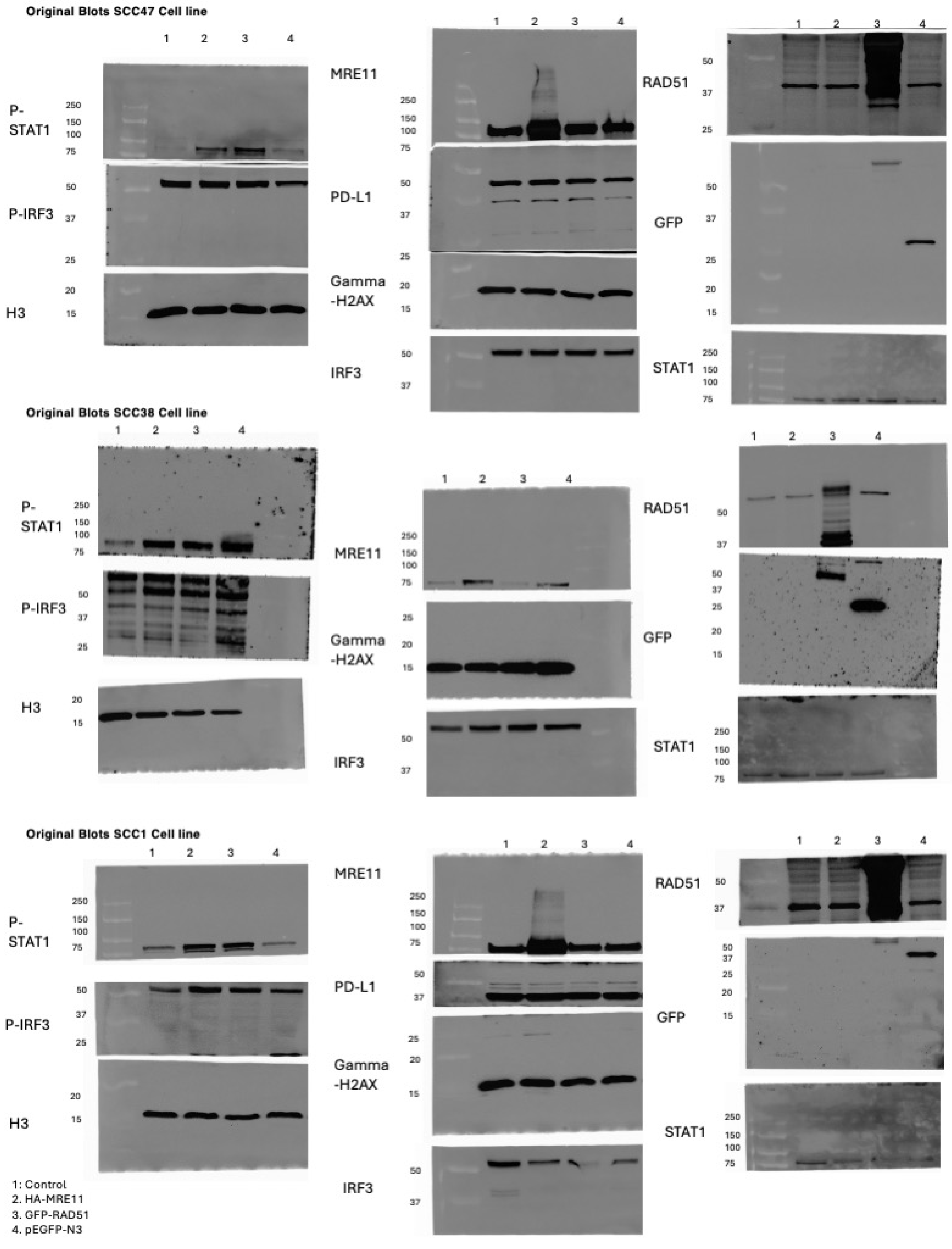

Figure S5. Uncropped and unprocessed scans of blotted images.
